# Evaluation of Microbial-Fructo-Oligosaccharides Metabolism by Human Gut Microbiota Fermentation as Compared to Commercial Inulin-Derived Oligosaccharides

**DOI:** 10.3390/foods11070954

**Published:** 2022-03-25

**Authors:** Dalila Roupar, Marta C. Coelho, Daniela A. Gonçalves, Soraia P. Silva, Elisabete Coelho, Sara Silva, Manuel A. Coimbra, Manuela Pintado, José A. Teixeira, Clarisse Nobre

**Affiliations:** 1 CEB—Centre of Biological Engineering, Campus de Gualtar, University of Minho, 4710-057 Braga, Portugal; dalila.roupar@ceb.uminho.pt (D.R.); mcoelho@ucp.pt (M.C.C.); daniela.goncalves@ceb.uminho.pt (D.A.G.); jateixeira@deb.uminho.pt (J.A.T.); 2 LABBELS-Associate Laboratory, 4710-057 Braga, Portugal; 3 CBQF—Centro de Biotecnologia e Química Fina—Laboratório Associado, Escola Superior de Biotecnologia, Universidade Católica Portuguesa, Rua Diogo Botelho 1327, 4169-005 Porto, Portugal; sara.nc.silva@gmail.com (S.S.); mpintado@ucp.pt (M.P.); 4 LAQV-REQUIMTE, Department of Chemistry, University of Aveiro, 3810-193 Aveiro, Portugal; soraiapiressilva@ua.pt (S.P.S.); ecoelho@ua.pt (E.C.); mac@ua.pt (M.A.C.)

**Keywords:** *Aspergillus ibericus*, prebiotics, glycosidic linkage analysis, neoFOS, short-chain fatty acids, qPCR

## Abstract

The prebiotic potential of fructo-oligosaccharides (microbial-FOS) produced by a newly isolated *Aspergillus ibericus*, and purified by *Saccharomyces cerevisiae* YIL162 W, was evaluated. Their chemical structure and functionality were compared to a non-microbial commercial FOS sample. Prebiotics were fermented in vitro by fecal microbiota of five healthy volunteers. Microbial-FOS significantly stimulated the growth of *Bifidobacterium* probiotic strains, triggering a beneficial effect on gut microbiota composition. A higher amount of total short-chain fatty acids (SCFA) was produced by microbial-FOS fermentation as compared to commercial-FOS, particularly propionate and butyrate. Inulin neoseries oligosaccharides, with a degree of polymerization (DP) up to 5 (e.g., neokestose and neonystose), were identified only in the microbial-FOS mixture. More than 10% of the microbial-oligosaccharides showed a DP higher than 5. Differences identified in the structures of the FOS samples may explain their different functionalities. Results indicate that microbial-FOS exhibit promising potential as nutraceutical ingredients for positive gut microbiota modulation.

## 1. Introduction

A growing interest in the consumption of prebiotics and in the understanding of their associated benefits to human health has been emerging in recent years. Prebiotics are food ingredients/components that can be selectively fermented by gut microbiota and, consequently, modify their composition, leading to an increased growth of bacteria beneficial to human health [1]. To create evidence of their role in a host’s health and disease—as well as unveiling the underlying metabolic mechanisms—multiple colon models have been developed over the past three decades [2].

Batch fermentations are the most common and simple in vitro models for studying the microbiota of the human gut. Batch fermentation models have been used to study the prebiotic potential of dietary components such as inulin-type fructans, resistant starch, and other complex carbohydrates [3,4,5,6]. They are mostly used to evaluate the metabolization of these specific substrates by selected isolated strains or complex fecal microbial communities, and to study its impact on the gut microbiota’s physiology and biodiversity [2]. The main disadvantages of batch fermentations are the rapid substrate depletion, the high accumulation of microbial metabolites, and the medium acidification, which prevents further microbial activity and operation under longer operation times. Nonetheless, in short-term fermentations, metabolites produced by some species become the substrate of other species, which may prevent substrate depletion [2]. Batch in vitro models provide a quick assembling, inexpensive running, easy operation, and reproducibility, allowing large numbers of substrates and/or fecal samples to be tested quickly [7,8]. The high throughput features make them almost an initial mandatory method to investigate gut microbiota composition, metabolism, and modulation by probiotics and diet compounds [2].

Fructo-oligosaccharides (FOS) are a recognized class of prebiotics and have been shown to promote a positive impact on gut microbiota composition and metabolic activity [9]. FOS can be found in many fruits and vegetables, which include pear, watermelon, artichoke, onion, nectarine, leeks, and garlic, among many others [10]. The production of FOS is mainly accomplished by inulin hydrolysis from natural sources. Enzymatically, FOS can be synthesized by transfructosylation of sucrose by fructosyltransferase enzymes that can be found in several microorganisms, mostly fungi, specifically *Aureobasidium pullulans* (a yeast-like fungus) [11], *Aspergillus* spp. [12], and *Penicillium* spp. [13]. Structurally, FOS are linear chains of fructose units linked by (β2→1)-glycosidic bonds with a single terminal glucose molecule linked to a fructose unit by an (α1→ β2)-linkage [14].

Because of their (β2→1)-fructose linkages, FOS reach the large intestine and the caecum intact, where they are fermented by the gut microbiota, resulting in changes in the bacterial communities’ composition and, consequently, in their metabolic activities [15]. FOS consumption by probiotic bacteria can strategically modulate the gut microbiota, resulting in an increased production of short-chain fatty acids (SCFA), contributing to the reduction of the luminal colon pH, suppressing the growth of enteric pathogens, and causing a protective effect on the intestinal barrier [16].

*Bifidobacterium* and *Lactobacillus* are considered among the most important genera in the gut microbiota due to their probiotic activities and susceptibility to the action of prebiotics [17]. Changes in the abundance of *Bifidobacterium* by the influence of prebiotics are more likely to be seen when compared to *Lactobacillus*, which may be explained by the highest preference of *Bifidobacterium* for oligosaccharides and its higher prevalence in the human gut [17]. Prebiotics can regulate the ratio between Firmicutes (the main phyla found in the human gut microbiota, which includes *Lactobacillus*) and Bacteroidetes [18]. They also impact the amount of Proteobacteria and Actinobacteria (where *Bifidobacterium* can be found) [9], which are of primordial importance for the well-being of the individual, since they can prevent the development of several diseases, and play an important role in the maintenance of the immunological activity [19].

Among the most produced SCFA are acetate, propionate, and butyrate, whilst branched-chain fatty acids (BCFA; isobutyrate, valerate, and isovalerate) and organic acids (lactate, succinate, and formate) are formed in a lower amount [9]. The source of the substrate, the microbiota composition, and the gut transit time all influence the SCFA production [20]. Acetate is produced at higher levels than other end-products of the gut microbiota, representing more than half of the total SCFA found in feces. It is mostly produced by enteric bacteria, namely Bacteroidetes and *Bifidobacterium,* and is rapidly absorbed in the proximal colon and further metabolized in the muscle, kidney, heart, or brain [21]. Propionate’s production is mainly associated with the *Bacteroides* via two main pathways: the fixation of carbon dioxide to form succinate and derived from lactate and acrylate. Although propionate is used as a minor source of energy for colonocytes, it is well known for its interaction with the immune system, which has an important anti-inflammatory effect [22]. Butyrate holds an important role in human health since it is used as a preferential energy source by the gut mucosa and has been related to anti-inflammatory and anti-carcinogenic properties. The production of butyrate is usually linked with Firmicutes phylum, including species of *Clostridium* [16].

Recent studies identified a newly isolated strain of *Aspergillus ibericus* as a good FOS producer [12]. FOS produced by the *A. ibericus* proved to have a bifidogenic effect, to promote the growth of *Lactobacillus,* and to be hydrolytic resistant to the harsh conditions of the gastrointestinal tract [23]. To increase the content and purity of the obtained FOS mixtures after *A. ibericus* fermentation, new integrated fermentative strategies have been developed [11,24]. In a previous study, FOS were produced by *A. ibericus* in co-culture with a *Saccharomyces cerevisiae* YIL162 W, and were able to metabolize non-prebiotic sugars for simultaneous FOS production and purification [24].

In the present work, microbial-FOS produced by the *A. ibericus* in co-culture with *S. cerevisiae* YIL162 W were characterized by gas chromatography-quadrupole mass spectrometry (GC-qMS), and their structure was further assessed by glycosidic linkage analyses. The microbial-FOS prebiotic potential was assessed by batch fermentation with human fecal inoculum, on the basis of (i) carbohydrate consumption, (ii) SCFA production, and (iii) gut microbiota composition modulation. Results were compared with a commercial non-microbial FOS sample (Raftilose^®^ P95).

## 2. Materials and Methods

### 2.1. FOS Samples

Microbial-FOS samples were produced using an integrated fermentation strategy previously reported by Nobre et al. [24]. A co-culture of *A. ibericus* with an *S. cerevisiae* YIL162 W was used, for simultaneous FOS production and purification by each strain, respectively. After fermentation, FOS mixture was desalted with activated charcoal in a shake flask, as described by Nobre et al. [25], with few modifications. Briefly, 40 mL of fermentative broth mixture was treated with 10 g of activated charcoal for 3 h at 165 rpm and 25 °C. Next, small sugars were removed by washing the activated charcoal three times with 150 mL of ultra-pure water. FOS were desorbed with 100 mL of ethanol (50% v/v) for 1 h, at 165 rpm and 25 °C. After a filtration step to remove the activated charcoal, a rotavapor (B. Braun Biotech International) was used to remove the ethanol and concentrate the carbohydrates by evaporation of the samples at 60 °C. Finally, the carbohydrates were freeze-dried (Heidolph lyophilizer).

Raftilose^®^ P95, a commercial FOS powder sample, was obtained from Beneo-Orafti Group (Oreye, Belgium).

### 2.2. Microbial-FOS Chemical Characterization

Microbial-FOS samples were identified and quantified after derivatization in alditol acetates [23]. The alditol acetates were dissolved in anhydrous acetone and analyzed by gas chromatography-quadrupole mass spectrometry (GC-qMS) according to an adaptation of the previously described methods [13,23] allowing the identification of FOS with higher DP. Briefly, the high-temperature capillary column HT5 (Trajan Scientific, Victoria, Australia) was used with 30 m of length, 0.25 mm of internal diameter, and 0.10 μm of film thickness. The injector port was equipped with a high-temperature inlet, septum, and graphite o-ring, and set at 400 °C. The chromatographic separation of derivatized FOS was attained with the initial temperature of 140 °C increasing 5 °C/min reaching 180 °C, holding for 1 min, after with a rate of 5 °C/min to 250 °C, holding for 10 min, and the final rate was set at 5 °C/min to 380 °C, holding for 10 min. For quantitative analysis, relative response factors of glucose, sucrose, 1-kestose, and stachyose to 2-deoxyglucose were determined based on five replicate analyses.

The glycosidic linkages of the FOS sample were identified by methylation analysis. FOS were methylated using CH_3_I, hydrolyzed (TFA 0.5 M) and the resultant monosaccharides were reduced (NaBD_4_) and acetylated. The partially methylated alditol acetates (PMAAs) obtained were analyzed by GC-qMS, as previously described [26]. 1-Kestose standard was used to normalize the terminally linked fructose, due to the lability of this residue in acidic media (correction factor: 0.68).

### 2.3. In Vitro Fecal Fermentation Assays

#### 2.3.1. Fecal Inocula Collection and Preparation

Fresh feces were provided from three male and two female healthy donors, with ages between 23 and 39 years old. An informed consent form was distributed among the un-related anonymous donors to provide the participant’s information about the study and the consent certificate assigned for each. Participants had not received antibiotic treatment, nor probiotic or prebiotic supplementation for at least 6 months before the fecal sample donation and had no history of gastrointestinal diseases. The fecal samples were collected into sterile flasks, maintained under anaerobic conditions, and used within a maximum 2 h after collection. The fecal inoculum (FI) was prepared following the methodology previously described [27]. Briefly, the FI was prepared by diluting the feces in a Reduced Physiological Salt solution (RPS). The RPS solution was constituted by cysteine-HCl (0.5 g/L) and NaCl (8.5 g/L) to obtain a concentration of 100 g feces/L RPS with a final pH of 6.8, in an anaerobic workstation acquired from Don Whitley Scientific (West Yorkshire, UK) with an atmosphere composed of 85% N_2_, 10% CO_2_, and 5% H_2_. The reagents cysteine-HCl and NaCl were obtained from Merck (Darmstadt, Germany) and LabChem (Zelienople, PA, USA), respectively.

#### 2.3.2. Fermentation Media Preparation

Fecal fermentations were performed with a Nutrient Base Medium. This medium was constituted by trypticase soy broth (TSB) without dextrose, and bactopeptone, in a concentration of 5.0 g/L, respectively. Cysteine-HCl was added at a concentration of 0.5 g/L. This medium was also composed of 0.2% (*v*/*v*) of a 0.5 g/L resazurin solution prepared in distilled water, 1.0% (*v*/*v*) of each trace minerals solution, and salt solution A. Salt solution B was added in the concentration of 0.2% (*v*/*v*). The salt solution A was constituted of 100.0 g/L NH_4_Cl, 10.0 g/L MgCl_2_·6H_2_O, 10.0 g/L CaCl_2_·2H_2_O while salt solution B was composed of 200.0 g/L K_2_HPO_4_·3H_2_O [28].

TSB and bactopeptone were purchased from Fluka Analytical (St. Louis, Missouri, EUA), and Becton Dickinson Biosciences (Franklin Lakes, NJ, USA), respectively. The reagents resazurin solution, trace minerals solution, and CaCl_2_·2H_2_O were acquired from Sigma-Aldrich Chemistry (St. Louis, Mo., USA), ATCC (Manassas, VA, USA), and Carlo Erba (Chaussée du Vexin, France), respectively. The cysteine-HCl, NH_4_Cl, MgCl_2_·6H_2_O, and K_2_HPO_4_·3H_2_O were obtained from Merck (Darmstadt, Germany). The final pH of the medium was adjusted to 6.8 with HCl (1 M) and then bubbled with N_2_ until a translucent/yellowish color. Next, 50 mL of basal medium was added to airtight anaerobic bottles, which were sealed with aluminum caps before sterilization by autoclave [28].

After sterilization, several carbon source solutions (sterilized by filtration through acetate cellulose syringe filters, with a 0.22 μm pore size) were added to the basal medium at 20 g/L final concentration, namely (i) the microbial-FOS sample produced by the *A. ibericus* in co-culture with the *S. cerevisiae*, (ii) a commercial FOS sample derived from inulin-Raftilose^®^ P95 (Beneo-Orafti, Belgium), and (iii) a solution without any added carbon source—negative control. A gas combination of 85% N_2_, 10% CO_2_, and 5% H_2_ was then used to reflux the basal medium to assure anaerobic conditions [28].

#### 2.3.3. Fecal Fermentations

Each fecal inoculum was added in a concentration of 2% (*v*/*v*) to the airtight anaerobic bottles previously prepared and incubated at 37 °C for 24 h without shaking. All manipulations were executed in an anaerobic workstation with an atmosphere composed of 85% N_2_, 10% CO_2_, and 5% H_2_. Samples were collected at 0, 12, and 24 h fermentation [28].

Aliquots of 4 mL of each collected sample were centrifuged at 4000 rpm for 6 min. The supernatants were utilized for carbohydrate and organic acid analysis, while the resulting pellet was used to obtain the genomic DNA for further bacterial population analysis [28].

### 2.4. Bacterial Population Analysis

#### 2.4.1. DNA Extraction

Genomic DNA (gDNA) was extracted and purified from human fecal samples using a NZY Tissue gDNA Isolation kit, according to the manufacturer’s protocol with some alterations. Briefly, pellets were homogenized in Tris Ethylenediamine Tetra Acetic Acid (Tris-EDTA) buffer (10 mM Tris/HCl; 1 mM EDTA; pH 8.0) and centrifuged at 4000 *g* for 10 min. This step was repeated until the supernatant was colorless. Afterward, 180 μL of a lysozyme solution (10 mg/mL lysozyme in a NaCl-EDTA solution (30 mM:10 mM)) was added and incubated at 37 °C for 1 h, with periodic shaking. Then, the samples were resuspended in 350 μL of NT1- buffer and incubated at 95 °C for 10 min. After this incubation step, samples were centrifuged at 11,000 *g* for 10 min at 4 °C, and 200 μL of supernatants were mixed with 25 μL of proteinase K and incubated at 56 °C for 1 h [28]. The following steps were executed according to the manufacturer’s protocol. After extraction, the gDNA’s concentration and purity were assessed through a NanoDrop spectrophotometer (Thermo Scientific, Wilmington, DE, USA).

#### 2.4.2. Quantitative Real-Time Polymerase Chain Reaction

Purified bacterial gDNA was detected and amplified by quantitative real-time PCR (qPCR) using a CFX96 Touch™ Real-Time PCR Detection System (Bio-Rad Laboratories, Inc., Hercules, CA, USA), as previously described [29].

qPCR reactions mixtures (total volume of 10 μL) included a volume of 5 μL of 2× iQTM SYBR^®^ Green Supermix, 1 μL of gDNA sample (equilibrated to 20 ng/µL), 1 μL of forward and reverse primers (100 nM) targeting the 16S rRNA gene, and 2 μL of ultra-pure water. The primer sequences used in this study were obtained from STABvida (Lisbon, Portugal) and are reported in Table S1, as well as the specific annealing temperature [29].

The amplification program consisted of one initial activation cycle of 95 °C for 10 min, followed by a denaturation step with 45 cycles at 95 °C for 10 s, an annealing step with 45 or 50 or 55 °C for 60 s according to each primer specific temperature, and an extension step at 72 °C for 15 s [6].

A melting curve analysis was performed for each PCR to ensure the specificity of the amplification by monitoring SYBER Green fluorescence in the temperature interval from 60 to 97 °C, with an increase of 0.1 °C (per 0.01 min). Data were processed and analyzed using the LightCycler software obtained from Roche Applied Science [28].

The most abundant phyla and genera in the healthy human gut microbiota (Firmicutes, *Clostridium leptum* subgroup, Bacteroidetes, and *Bacteroides*), as well as recognized probiotics (*Bifidobacterium* and *Lactobacillus*), were selected as target groups. All analyses were carried out in quadruplicate, and standard curves were calculated using tenfold bacterial dilution gDNA standards of *Clostridium leptum* (ATCC 29065), *Bacteroides vulgatus* (ATCC 8482), *Bifidobacterium longum* subsp. *infantis* (ATCC 15697) (DSMZ, Braunschweig, Germany), and *Lactobacillus gasseri* (ATCC 33323) (Table S1). In this study, the NCBI Genome database was used to obtain the genome size for each bacterial strain used as a standard, as well as the copy number of the 16S rRNA gene (www.ncbi.nlm.nih.gov, accessed on 15 December 2021).

### 2.5. Carbohydrate and Fermentation Product Analysis

As a first step, the fermentation supernatants were filtered using a 0.22 μm acetate cellulose syringe filter. Afterward, sugars and relevant SCFA (acetate, propionate, and butyrate) concentrations were calculated by high-performance liquid chromatography (HPLC).

For sugar analysis, an HPLC system from Jasco (Tokyo, Japan) was used. Briefly, this HPLC system was equipped with a Prevail Carbohydrate ES column (5 μm, 25 cm × 0.46 cm length × diameter) (Alltech) and a refractive index detector. The fecal samples were eluted with a mixture of acetonitrile (HPLC Grade, Fisher Chemicals, Belgium) and pure water (70:30 (*v*/*v*)), and 0.04% of ammonium hydroxide (HPLC Grade, Sigma-Aldrich, St. Louis, MO, USA) in water. The elution was carried out at a flow rate of 1 mL/min and 30 °C [24]. The chromatographic signal was registered and then integrated using the Star Chromatography Workstation 6.3 software (Varian, CA, USA). FOS standards (1-kestose (GF_2_), nystose (GF_3_), and fructo-furanosylnystose (GF_4_)) were obtained from Wako (Japan). Sucrose and fructose standards were acquired from Merck and glucose from VWR (Belgium).

For the quantification of lactic acid and SCFA, an HPLC system equipped with an Aminex HPX-87H chromatographic column (8 µm, 30 cm × 0.46 cm length × diameter) coupled with a UV detector (wavelength: 210 nm) was used. Samples were eluted in a solution of sulfuric acid (5 mM) at a 0.7 mL/min flow rate and 60 °C [30]. The chromatographic signal was recorded and further integrated using the Star Chromatography Workstation 6.3 software (Varian, CA, USA). SCFA and lactic acid standards were obtained from Merck.

### 2.6. Statistical Analysis

The statistical analysis was carried out using GraphPad Prism 8.2.1 (San Diego, CA, USA). The normality of the data’s distribution was evaluated through Shapiro–Wilk’s test. As the data proved to follow a normal distribution, One-way ANOVA, coupled with Tukey’s post hoc test or the Student’s *t*-test, was used to determine significant differences in the quantification of bacteria populations by qPCR, at each time point. Repeated Measures ANOVA was used to evaluate the bacterial population over time. Differences were considered significant for *p*-values ≤ 0.05.

## 3. Results

### 3.1. Microbial-FOS Chemical Characterization

The composition of the microbial-FOS mixture obtained in co-culture with the *S.* *cerevisiae* YIL162 W strain is described in Table 1. Nystose (GF_3_) was the fructo-oligosaccharide produced in higher amounts (495.8 mg/g of purified sample), followed by 1-kestose (204.6 mg/g). These FOS have an inulin structure, which consists of a linear chain of (β2→1)-fructose residues linked to a sucrose moiety. The identification of these structures is supported by glycosidic linkage analysis (Table S2), as terminally linked fructose (*t*-Fru), (2→1)-Fru, and terminally linked glucose (*t*-Glc) were the residues found in higher proportions. In addition, the high amount of *t*-Fru and *t-*Glc (30% and 24%, respectively) indicates the presence of fructo-oligosaccharides with a low polymerization degree. The FOS mixture composition showed that 87% of the identified carbohydrates had a degree of polymerization (DP) inferior or equal to 5 (Table 1).

The residue (1→6)-Glc (3%) suggests the presence of oligosaccharides from the inulin neoseries type, such as neokestose (8.3 mg/g). Furthermore, the compound eluted after nystose (Figure S1), assigned also as DP 4 (27.0 mg/g), had a mass spectrum similar to that of neokestose and can correspond to 1^F^,6^G^-Di-β-d-fructofuranosylsucrose or 6^G^(1-β-d-fructofuranosyl)_2_-sucrose, which are classified as neoFOS inulin type. 1^F^-β-Fructofuranosylnystose (GF_4_) accounted for 76 mg/g, in equivalents of stachyose (DP 4). This could result in an underestimation of the GF_4_ quantity, as the GC-qMS sensitivity decreases inversely with increasing DP. The remaining residues found in smaller proportions (6-Fru, 1,6-Fru, and 4-Glc) can be ascribed to other tri- and tetrasaccharides that were below the limit of quantification, such as levan and/or mixed-type FOS with a DP higher than 5, which accounted for about 13% of the sample (Table 1).

On the other hand, Raftilose^®^ P95, structurally characterized in a previous work, was richer in inulobiose and inulotriose and did not contain neoseries sugars (Table S3). In addition, this commercial FOS had a higher average DP (2–7) and was mainly composed of tri-, tetra-, and penta-oligomers (Table S4) [23].

### 3.2. Fermentation with Human Gut Microbiota

#### 3.2.1. Carbohydrate Consumption

**Figure 1:**
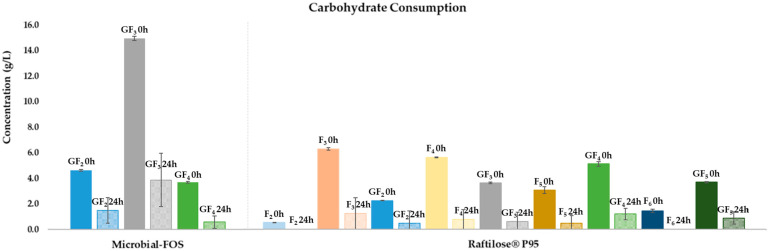
Carbohydrate profiles of microbial-fructo-oligosaccharides (Microbial-FOS) and Raftilose^®^ P95, before and after 24 h fermentation with fecal microbiota from five donors. Values are expressed as mean ± SD. GF_2_—1-kestose, GF_3_—nystose, GF_4_—fructo-furanosylnystose, F_n_ (2 < *n* < 6)—FOS.

Both microbial-FOS and Raftilose^®^ P95 were extensively metabolized by the gut microbiota of the five human healthy donors but showed differences in their consumption pattern. Figure 1 shows the concentration profile of each individual sugar present in the microbial-FOS and Raftilose^®^ P95 samples, before inoculation (0 h) and after 24 h of fermentation with the microbiota from the five donors. At the beginning of fermentation, the microbial-FOS mixture contained in the basal medium included 23.9 ± 0.4 g/L oligosaccharides and 1.9 ± 0.1 g/L smaller carbohydrates, while Raftilose^®^ P95 held 31 ± 0.9 g/L and 1 ± 1 g/L, respectively.

After 24 h of fermentation, the amount of oligosaccharides in the microbial-FOS decreased to 10 ± 3 g/L and in Raftilose^®^ P95 to 8 ± 5 g/L, representing a metabolization of 62 ± 10% and 76 ± 17% oligosaccharides, respectively.

**Figure 2:**
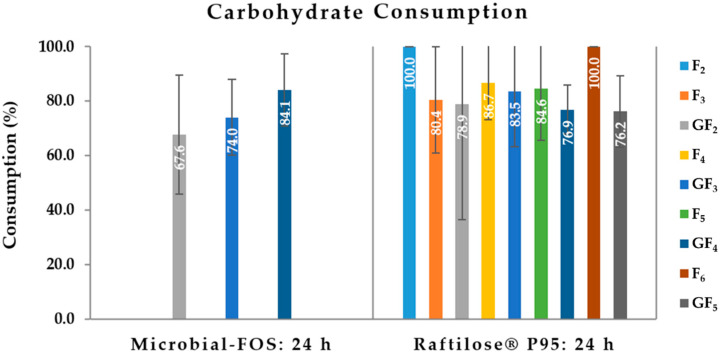
Consumption profile of each carbohydrate present in the microbial-fructo-oligosaccharides (Microbial-FOS) and Raftilose^®^ P95 sample, after 24 h fermentation with fecal microbiota from five donors. GF_2_—1-kestose, GF_3_—nystose, GF_4_—fructo-furanosylnystose, F_n_ (2 < *n* < 6)—FOS.

The microbiota was able to metabolize all carbohydrate types contained in the mixtures. Up to 84 ± 13% GF_4_ were consumed, followed by 74 ± 14% GF_3_, and finally 68 ± 22% GF_2_ (Figure 2). For Raftilose^®^ P95, at least 76% of each individual sugar was consumed, and F_2_ and F_6_ were totally metabolized by the microbiota (Figure 2).

#### 3.2.2. Human Gut Microbiota Composition

**Figure 3:**
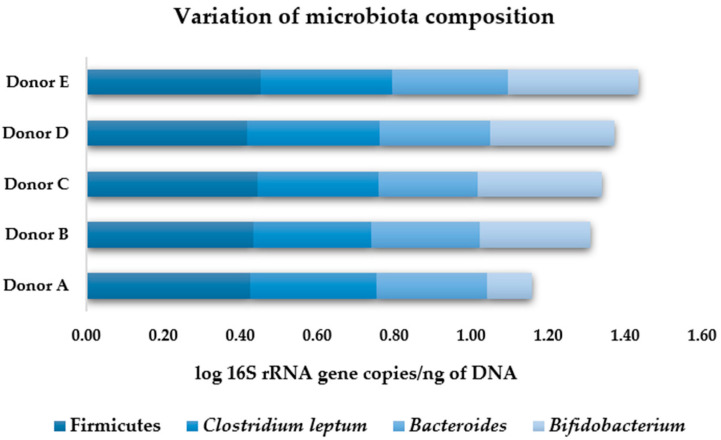
Overall variation of the distinct bacterial groups for each donor at 0 h fermentation time. Values are expressed as log 16S rRNA gene copies/ng of DNA.

**Figure 4:**
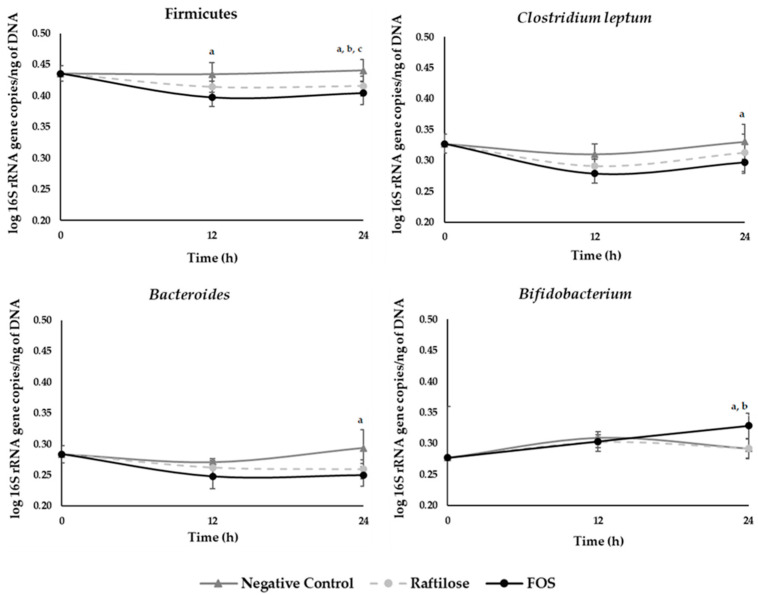
Variation of Firmicutes, *Clostridium leptum*, *Bacteroides,* and *Bifidobacterium* from five microbiota donors during fermentation with microbial-fructo-oligosaccharides (FOS), Raftilose^®^ P95 and without carbon source (negative control). Values are expressed as log 16S rRNA gene copies/ng of DNA through time (h), with standard deviation bars; a, b, c means statistically significant differences (*p* < 0.05) between: a—microbial-FOS and negative control, b—microbial-FOS and Raftilose^®^ P95, c—Raftilose^®^ P95 and negative control.

To quantify changes in the bacterial population of the gut microbiota, the real-time polymerase chain reaction (qPCR) method with 16S rRNA-based specific primers [22] was used. Figure 3 illustrates the bacterial groups identified in each donor at 0 h fermentation time and Figure 4 shows the microbiota composition, on average copy numbers, obtained by qPCR of the bacteria main groups analyzed throughout the fermentation. Bacteria that belong to the three dominant phyla in the human gut were evaluated: Firmicutes (Firmicutes phyla and *Clostridium leptum* subgroup), Bacteroidetes (*Bacteroides*), and Actinobacteria (*Bifidobacterium*). 

Similar microbiota composition was determined for all participants, with exception of donor A, for which the abundance of the *Bifidobacterium* genus was lower than the other donors (Figure 3).

The human microbiota collected before fermentation was mainly composed by bacteria from the Firmicutes group (0.44 ± 0.01 log 16S rRNA gene copies/ng of DNA), followed by the *Clostridium leptum* subgroup (0.33 ± 0.02 log 16S rRNA gene copies/ng of DNA), and in similar proportion *Bacteroides* (0.28 ± 0.01 log 16S rRNA gene copies/ng of DNA) and *Bifidobacterium* (0.28 ± 0.08 log 16S rRNA gene copies/ng of DNA) (Figure 4).

Figure 4 shows the microbiota growth dynamics after supplementation with microbial-FOS, Raftilose^®^ P95, and without carbon source (negative control). Regarding the first 12 h of fermentation, *Bacteroides* and *Bifidobacterium* did not grow (*p* > 0.05) and there was a significant decrease of *Clostridium leptum* (*p* < 0.05) for all tested conditions, while Firmicutes phylum growth remained similar with Raftilose^®^ P95 and significantly decreased with the microbial-FOS.

After 12 h of fermentation and up to 24 h, Firmicutes, *Clostridium leptum,* and *Bacteroides* did not grow (*p* > 0.05) for all tested conditions, regardless of the carbon source used. However, a significant growth for *Bifidobacterium* was observed between 12 h and 24 h of fermentation when using microbial-FOS (*p* < 0.05).

Comparing the three different tested conditions, the fecal samples with microbial-FOS had lower microbial populations than the negative control for Firmicutes, *Clostridium leptum*, and *Bacteroides* at 24 h (*p* < 0.05). Regarding Raftilose^®^ P95, only the Firmicutes phylum had a lower population than the negative control (*p* < 0.05) at 24 h. *Clostridium leptum* and *Bacteroides* profiles were similar to the negative control. It was also possible to observe that the number of gene copies for Firmicutes in microbial-FOS fermentation was lower than Raftilose^®^ P95 samples at 24 h (*p* < 0.05).

Interestingly, despite no growth effect being found for microbial-FOS nor Raftilose^®^ P95 concerning the Firmicutes group, the *Clostridium leptum* subgroup and the *Bacteroides* group achieved a growing trend from 12 h to 24 h of fecal fermentation when compared to the negative control (*p* > 0.05). This might indicate that whereas *Bifidobacterium* initiates the fermentation process, the remaining bacterial communities have a delayed growing dynamic, which is consistent with their growth over the 12 to 24 h period.

#### 3.2.3. Short-Chain Fatty Acid Production

The variation on the SCFA profile, namely acetate, propionate, and butyrate, and on the organic acids lactate, succinate, and formate during microbiota fermentation with microbial-FOS, Raftilose^®^ P95, and without carbon source (negative control) is shown in Table 2.

In this work, acetate, propionate, butyrate, lactate, succinate, and formate were produced by fermentation of the microbial-FOS. At 24 h of fermentation, there was a higher production of acetate for both microbial-FOS and Raftilose^®^ P95 when compared to the negative control sample (*p* < 0.05). After 12 h of fermentation, microbial-FOS already had an increasing amount of acetate formed (*p* < 0.05). No significant differences were observed between fecal fermentations with microbial-FOS and Raftilose^®^ P95.

Higher propionate production was also observed for fecal fermentations with microbial-FOS and Raftilose^®^ P95 when compared to the negative control samples at both 12 h and 24 h (*p* < 0.05). Microbial-FOS fermentation led to a higher propionate production than Raftilose^®^ P95 at 12 h (*p* < 0.05), but no significant difference was found at 24 h.

Regarding butyrate, a strong butyrogenic effect was found in the fecal fermentations with microbial-FOS, as its production was higher than both Raftilose^®^ P95 and negative samples at 24 h (*p* < 0.05 for both). In addition, higher butyrate production was observed for microbial-FOS samples when compared with Raftilose^®^ P95 samples at 12 h (*p* < 0.05).

Lactate concentration increased over the entire fecal fermentation for both microbial-FOS and Raftilose^®^ P95 (*p* < 0.05 for both between 0 h and 24 h), although for the negative control samples the lactate concentration decreased over time (*p* < 0.05). Nonetheless, it did not show a statistically significant variation between 12 and 24 h for the microbial-FOS (*p* > 0.05), whereas it increased for Raftilose^®^ P95 (*p* < 0.05). This was followed with a consistent trend in propionate and butyrate production, which was maintained with microbial-FOS between 12 and 24 h, while decreased with Raftilose^®^ P95 (*p* < 0.05).

Considering the total amount of SCFAs, a higher production was observed in microbial-FOS (5.00 ± 0.37 g/L at 12 h and 5.29 ± 0.48 g/L at 24 h) and Raftilose^®^ P95 fermentations (4.04 ± 0.48 g/L at 12 h and 4.18 ± 0.76 g/L at 24 h), as compared to the negative control (2.50 ± 0.68 g/L at 12 h and 2.62 ± 0.50 g/L at 24 h) (*p* < 0.05). In addition, at 12 h of fermentation, the total amount of SCFA was statistically higher in samples from microbial-FOS than in Raftilose^®^ P95 ones (*p* < 0.05). Regarding the SCFA concentration ratios, an acetate:propionate:butyrate ratio of 1.3:0.6:1.0 was obtained for the negative control, 2.1:1.5:1.0 for Raftilose^®^ P95 and 1.1:1.1:1.0 for microbial-FOS.

## 4. Discussion

Microbial-FOS produced by *A. ibericus* in mono-culture were structurally characterized in a previous work [12]. In mono-culture fermentation, glucose accounted for more than 27% (*w*/*w*) of the total sugars at the optimal harvest point, increasing the caloric value of the mixture and, consequently, decreasing its prebiotic functionality [12]. The high amount of glucose released during the fermentation also inhibited the transfructosylation enzymatic reaction, resulting in a mixture always containing non-reacted sucrose.

Envisaging the production of FOS mixtures with higher purity and content, FOS were produced in a previous work using an integrated fermentation with co-culture of *A. ibericus* with *S. cerevisiae* YIL162 W [24]. The yeast contains the gene responsible for sucrose hydrolysis disrupted, avoiding competition by the substrate with the fungi, and was able to remove the non-prebiotic sugars released during fermentation. The elimination of glucose resulted in lower non-reacted amount of sucrose, which decreased by 54% when compared to the FOS mixture produced in mono-culture (Table 1 and Table S5). Thus, FOS were the predominant acceptors of the fructose moiety, increasing the amount of nystose and fructo-furanosylnystose by 68% and 210%, respectively.

In the present study, the prebiotic potential of FOS produced in the co-culture with *A. ibericus* and *S. cerevisiae* was assessed by fermentation with human gut microbiota, and compared with the prebiotic potential of a non-microbial commercial FOS sample. Raftilose^®^ P95 was chosen among the available commercial FOS samples, since its structure is similar to the microbial-FOS in terms of DP, and at the same time different because it is derived from inulin and not from sucrose [23]. The majority of the existing commercial FOS are derived from inulin (inulin-type), which is the case of Raftilose^®^ P95 and Inulin-S, Raftiline^®^ HP, Fibrulose^®^ F97, and Fibruline^®^ Instant [31,32]. Inulin-derived fructans may include FOS without a terminal glucose residue (F_n_ type), which are not found in microbial-FOS. Microbial-FOS are short oligomers synthesized from sucrose, with two to four fructose residues always linked to a terminal glucose unit (GF_n_ type) [23]. Instead, Raftilose^®^ P95 includes both types of FOS [31]. From the commercial samples identified, Raftilose^®^ P95 had the lowest DP (2–7). On the other hand, both Inulin-S and Fibruline^®^ Instant have a DP between 2–60; Raftiline^®^ HP an average DP > 23; and Fibrulose^®^ F97 a DP up to 20 [31,32]. For these reasons, Raftilose^®^ P95 was chosen as the commercial sample for assessing prebiotic effect as compared to the microbial-FOS. The influence of the structures of each type of FOS in the microbial growth and function was evaluated.

From the chemical characterization of the samples (Table 1) it was possible to identify GF_3_, followed by GF_2_ and GF_4_, as the main sugars composing the microbial-FOS, and in a smaller amount neokestose (n-GF_2,_ 6^G^(1-β-_D_-fructofuranosyl)sucrose) (Table S2). The production of neokestose and neonystose (n-GF_3_, 6^G^(1-β-_D_-fructofuranosyl)_2_sucrose) by other fungi strains have been reported, such as *Penicillium citrinum* [33] and *Xanthophyllomyces dendrorhous* [34]. Neokestose was also identified in a previous work conducted with *A. ibericus* in mono-culture [23].

On the other hand, Raftilose^®^ P95 was richer in inulobiose and inulotriose and did not contain neoseries sugars (Table S3). Inulin-derived fructans may include FOS without a terminal glucose residue (FF_n_ type) in its composition, which were not found in microbial-FOS [20]. Recently, inulobiose, inulotriose, inulotetraose, and nystose were identified in a Raftilose^®^ P95 sample by other authors [35]. Raftilose^®^ P95 also showed a higher DP (2–7) than the microbial-FOS, and was mainly composed of tri-, tetra-, and penta-oligomers (Table S4), which is also in accordance with the literature [35].

Results on the carbohydrate consumption and SCFA production after 24 h of fermentation demonstrated that both microbial-FOS and Raftilose^®^ P95 were suitable fermentable carbohydrates for the human gut bacteria. The studied gut microbiota was able to metabolize all fructo-oligomers (both GF_n_ and F_n_ type) present in the samples.

Long-chain oligosaccharides (GF_4_ > GF_3_ > GF_2_) from the microbial-FOS sample seem to be preferentially consumed by the microbiota, even if no statistical difference was found (*p* > 0.05). Nevertheless, there was no sugar preference pattern in the Raftilose^®^ P95 sample. It has been reported that each bacterial strain may exhibit distinct preferences for different chain lengths and types of FOS. For instance, *Lactobacillus plantarum* preferentially consumed GF_2_ and GF_3_ [36], whilst *Lactobacillus casei* 1134 and *Lactobacillus rhamnosus* 1136 only utilized GF_2_ [37]_._ Furthermore, many *Bacteroides* species can ferment GF_2_ and GF_3_, but no information regarding GF_4_ consumption was provided [37]. The microbiota used in this study have the ability to metabolize short-chain fructans, either those produced by microbial synthesis or extracted from inulin (Raftilose^®^ P95).

Regardless, most of the bacteria are able to consume short-chain carbon sources, but only a few can utilize long-chain fructans [38,39]. *Bifidobacterium*, *Bacteroides*, and *Lactobacillus* have been shown to ferment oligofructans (DP 2–7), including Raftilose^®^ P95 [36]. It has been reported that the majority of *Bifidobacterium* strains can utilize short-chain fructans, but not highly polymerized inulin [39,40]. In addition, short-chain fructans increased bifidobacteria to a higher extent than longer fructans [41]. On the other hand, no differences were observed for *Lactobacillus* in this matter. As perceived in this study, both microbial-FOS and Raftilose^®^ P95 were capable of successfully modulating the gut microbiota composition. More than 70% of each fructo-oligomer was consumed after 24 h, with the exception of the GF_2_ of the microbial-FOS. This resulted in the metabolization of >60% of the total oligosaccharides in both prebiotic samples.

Gut microbiota composition modulation by the carbohydrates was evaluated by qPCR method in the present study. The detection of groups, genera, or specific species within a complex bacterial population, such as human fecal samples, can be easily accomplished using this technique, which also has the advantage of being fast [42].

At 0 h of fecal fermentation, a similar microbiota composition was observed for all participants, except for the *Bifidobacterium* genus present in the fecal sample of donor A (Figure 3). This can be explained by the fact that each person’s microbiota profile is unique, and it is the result of an array of different factors [43]. As the set of donors used in the present study comprised both men and women of different ages, it is important to consider each volunteer’s unique microbial profile.

The human gut microbiota collected before fermentation was mainly composed of bacteria from the Firmicutes group, followed by the *Clostridium leptum* subgroup, and in similar proportion *Bacteroides* and *Bifidobacterium* (Figure 4). These findings are in accordance with the literature, which shows that Firmicutes and Bacteroidetes are the two dominant phyla of healthy gut microbiota and represent almost 80–90% of its bacterial community [44]. Regarding the dynamics of microbiota growth, the non-growth of the Firmicutes, *Clostridium leptum*, *Bacteroides,* and *Bifidobacterium* in the first 12 h of fermentation may be explained by association with an adaptive phase of the microorganisms to the fermentation medium, meaning no growth or even some death of cells [45]. In addition, in the last 12 h fermentation, Firmicutes, *Clostridium leptum,* and *Bacteroides* did not grow for all tested conditions. Since the experimental setup used did not enable continuous pH adjustments, this pattern could be associated with the fact that the acid compounds generated over the fermentation lead to a decrease in the pH, which may have inhibited the growth [38]. However, significant growth for *Bifidobacterium* was observed in fermentation samples with microbial-FOS. This highlights its bifidogenic effect, which was not observed with Raftilose^®^ P95, hence indicating a unique prebiotic effect of the *A. ibericus* microbial-FOS.

Nobre et al. [23] studied the effect of FOS produced by *A. ibericus* and *A. pullulans*, and Raftilose^®^ P95, on the growth of *Lactobacillus* and *Bifidobacterium* isolated strains. Overall, FOS synthesized by *A. ibericus* stimulated a higher growth of the *Lactobacillus* strains, followed by *A. pullulans* and Raftilose^®^ P95, but a similar growth of *Bifidobacterium* was obtained [23]. Nevertheless, results suggested that the fermentation of the carbohydrates depends on the bacterial strain used rather than on the species or genera. In another work, the impact of different oligosaccharides on the human gut microbiota modulation was studied [41]. Results shown that NutraFlora^®^ P95 (a microbial-FOS) had similar *Bifidobacterium* growth as compared to Beneo P95 (also known as Raftilose^®^ P95). In the present study, *A. ibericus* microbial-FOS stimulated a higher growth of *Bifidobacterium* strains, which may be related to the specific *Bifidobacterium* strains present in the gut microbiota under study, as well as on the microbiota consortium interaction.

The higher bifidogenic effect observed for the microbial-FOS samples, as compared to Raftilose^®^ P95, may also be related to their FOS structures, since only microbial-FOS has neokestose in its content [13,23]. Neokestose has been shown to induce higher growth of bifidobacteria and lactobacilli as compared to a commercial FOS, and inhibits the growth of pathogenic bacteria such as *Clostridium* and *Bacteroides* [46]. The neoFOS structure is very similar to inulin-type FOS (1^F^-FOS) since both have fructosyl units bound by a (β2→1)-linkages. However, (β2→6)-linkages between fructose and a glucosyl group (6^G^-FOS) are only present in the neoFOS series [47]. *Bifidobacterium* strains have been shown to efficiently transport and metabolize (β2→6)-FOS [48,49]. Furthermore, a higher growth of probiotic microorganisms was observed in the presence of (β2→6)-linked compounds than with other prebiotic substrates such as inulin and Actilight^®^ FOS [50]. NeoFOS have shown also higher pH resistance and thermal stability than inulin-type FOS [51]. Thus, better chemical stability may be one of the reasons for neoFOS to reach the colon almost unmodified, serving as a substrate for the intestinal microorganisms, inducing a higher bifidogenic effect. A correlation between (β2→6)-linked FOS length and higher resistance against acid or enzymatic hydrolysis has also been reported [48]. Herein, neoFOS up to DP 4 were identified in the microbial-FOS sample, which may enhance its bifidogenic effect. Moreover, the structure and/or degree of polymerization of the different FOS sources affect the individual strains’ lag phase, cell densities, and growth rates [5]. These parameters may explain the differences obtained on the gut microbiota population after supplementation with these two different FOS subtracts, and therefore this will be reflected in their potential prebiotic properties. At the end of the fecal fermentation, just samples with microbial-FOS had lower microbial populations for Firmicutes, *Clostridium leptum*, and *Bacteroides*. Other authors have reported that (β2→6)-linked oligosaccharides induce a decrease of the *Clostridium* group in fecal microbiota [52]. Hence, the presence of neoFOS in microbial-FOS could explain this outcome.

In this work, the prebiotic potential of microbial-FOS was also confirmed by the production of SCFA. Both microbial-FOS and Raftilose^®^ P95 have been found to lead to higher acetate production. This finding resonates with the underlying rationale that microbial-produced FOS can be used to stimulate the metabolic activity of the gut microbiota. Moreover, the higher acetate concentration in microbial-FOS samples could also be directly correlated with the strong bifidogenic effect found for the fecal samples with microbial-FOS (c.f. Table 2), as this bacterial group is recognized as the key acetate producer in the human gut [53]. Indeed, *Bifidobacterium* performs carbohydrate fermentation through hydrolysis of acetyl-CoA [22]. Another source of acetate production in the human gut is acetogenic bacteria that use hydrogen, carbon dioxide, or formate via the Wood–Ljungdahl pathway [16]. This is consistent with the observed correlation between acetate and formate concentrations since formate concentration decreases as the acetate concentration increases. However, since microbial-FOS present the lowest reduction in formate concentration, this seems to put in evidence that the significant increase in acetate as compared to the negative control is directly related to the strong bifidogenic effect found for the microbial-FOS. A more pronounced production of acetate when using neokestose as a substrate has already been reported [46]. In addition, a diet rich in (β2→6)-linked oligosaccharides also significantly increased the acetate levels in the colon of rats [54]. Hence, the higher values obtained could be related to the presence of neokestose on the microbial-FOS.

Propionate represents a minor source of energy for colonocytes, but it has well-known anti-inflammatory properties on human health [53]. The propionate production in the human gut is commonly associated with bacteria belonging to the Bacteroidetes phylum. Nonetheless, in this study, the *Bacteroides* population was found to be larger in the negative control, which conflicts with the observed higher propionate content. However, the production of propionate in the human gut is also linked to Verrucomicrobia and to bacteria belonging to the class *Negativicutes* [53]. In addition, propionate can be synthesized via three different metabolic pathways: acrylate, succinate, or propanediol [22]. Since succinate concentration remains significantly higher in microbial-FOS fecal samples, this could explain the higher propionate production in these samples due to the higher bioavailability of intermediate substrates, even considering the observed reduction of the *Bacteroides* population.

In addition, higher butyrate production was observed for microbial-FOS samples when compared with Raftilose^®^ P95. Despite no increase having been found for butyrate bacteria producers, such as Firmicutes and *Clostridium leptum* subgroup, the strong butyrogenic effect from microbial-FOS can be related to the capacity of Firmicutes species to convert lactate and acetate into butyrate through cross-feeding mechanisms [16,22,53]. These cross-feeding mechanisms between bifidobacteria and the acetate-dependent *Faecalibacterium prausnitzii* producer of butyrate have been reported [55]. Depending on the inulin degradation capacity of the bifidobacterial strains involved, these can be commensal interactions or subjected to competition for the available substrate [55]. Indeed, the fecal samples with microbial-FOS showed a strong bifidogenic effect, which is associated with a higher acetate production—whose increased bioavailability subsequently explains the higher butyrate production found. A positive correlation between acetate and butyrate production has also been found by Jang et al. [54].

Lactate concentration increased over the entire fecal fermentation for both microbial-FOS and Raftilose^®^ P95. These results are explainable via the aforementioned cross-feeding processes since lactate (already produced by other bacteria) can also be used as a substrate by bacteria producers of butyrate and propionate [16,22], preventing lactate accumulation and excess acidity [9]. Lactate levels were not detected after 72 h in a recent study by Liu et al. [52], corroborating the obtained results. The authors concluded that the lactate was consumed by butyrate-producing bacteria such as *Clostridium* spp. [52].

Moreover, the butyrate-producing bacteria are phylogenetically combined with non-butyrate producers, and consequently, its quantification with molecular techniques targeting the 16S rRNA gene becomes difficult [22]. Nonetheless, the observed data support the underlying rationale that microbial-FOS are able to generate a strong butyrogenic effect, which has the potential to trigger beneficial anti-inflammatory, anti-carcinogenic and satiety-inducing effects on their host [53].

Considering the total SCFAs, a higher production was observed in microbial-FOS as compared to the Raftilose**^®^** P95. Therefore, there is a very clear trend that supports the claim that microbial-FOS induces a stronger SCFA synthesis in the fecal sample fermentations undertaken in the present work. Regarding the SCFA concentration ratios, for microbial-FOS an acetate:propionate:butyrate ratio of 1.1:1.1:1.0 was obtained, and 2.1:1.5:1.0 for Raftilose**^®^** P95. Several authors have reported that polysaccharide fermentation frequently results in acetic, propionic, and butyric acids in a 3:1:1 ratio [16], but these proportions were not observed in the presented results. In particular, for microbial-FOS this might result from the strong butyrogenic effect found, which can be explained by cross-feeding interactions, where acetate is used by butyrate-producing bacteria, such as Firmicutes and Clostridial clusters, to form butyrate [53]. In addition, the (β2→6)-glycosidic bonds characteristic of neoFOS can only be cleaved by a few bacterial species, which can explain the differences between the obtained results [56].

The holistic analysis of the entire data acquired with this study puts in evidence how the differences in carbohydrate profiles from different FOS have a direct impact on the dynamics of the gut microbiota, namely distinct production of benefic SCFA. In particular, this underlying rationale is supported by the presence of a neoseries oligosaccharide, which has a bifidogenic effect demonstrated through an increase in the *Bifidobacterium* population and in acetate production. This is a very interesting finding, which was not previously found on tests conducted with isolated strains, highlighting the importance of the strong interplay within gut microbiota and the effects of cross-feeding mechanisms. This also emphasizes the pitfalls of testing novel gut-modulating nutraceuticals on consortiums of isolated strains, as their complex metabolic interactions play a central role.

## 5. Conclusions

The present study brings forward a first characterization of the prebiotic potential of a novel microbial-FOS produced by an *A. ibericus* in co-culture with an *S. cerevisiae* YIL162 W using human fecal samples.

Based on the structure analyses, there are significant differences in the structural composition of the microbial-FOS vs. Raftilose^®^ P95. Neoseries-type FOS, such as neokestose (DP 3), possibly neonystose (DP 4), and neofructo-furanosylnystose (DP 5), were identified in the microbial-FOS but not found in Raftilose^®^ P95. These structural differences may justify the strongest bifidogenic and butyrogenic effects found in fermentations run with the microbial-FOS. The microbial-FOS bifidogenic effect found in the present work, while fermented by human gut microbiota, reinforces the importance of cross-feeding mechanisms on the overall prebiotic evaluation of novel nutraceutical ingredients.

FOS produced by *A. ibericus* were found to successfully modulate gut microbiota and stimulate the SCFA production, providing a significantly higher concentration of these beneficial components than Raftilose^®^ P95. The further incorporation of the microbial-FOS in new formulations may result in excellent novel functional foods.

## Figures and Tables

**Table 1:** Carbohydrate composition (mg/g of purified sample) obtained for the microbial-FOS mixture produced by the co-culture between *A. ibericus* and *S. cerevisiae* YIL162 W.

Assignment		Microbial-FOS Mixture (mg/g)	RSD (%)
DP 1	Fructose	27.4	1
DP 2	Sucrose	32.2	16
DP 3	1-Kestose	204.6	13
	Neokestose	8.3	8
DP 4	Nystose	495.8	18
	1^F^,6^G^-Di-β-d-fructofuranosylsucrose or 6^G^(1-β-d-fructofuranosyl)_2-_sucrose	27.0	17
DP 5	1^F^-β-d-Fructofuranosylnystose	76.2	17
	1^F^-(1-β-d-fructofuranosyl)_2_-6^G^-β-d-fructofuranosylsucrose or 6^G^(1-β-d-fructofuranosyl)_3-_sucrose or 1^F^-1-β-d-fructofuranosyl-6^G^-(1-β-d-fructofuranosyl)_2-_sucrose	<LOQ	
	1^F^-(1-β-d-fructofuranosyl)_2_-6^G^-β-d-fructofuranosylsucrose or 6^G^(1-β-d-fructofuranosyl)_3-_sucrose or 1^F^-1-β-d-fructofuranosyl-6^G^-(1-β-d-fructofuranosyl)_2-_sucrose	<LOQ	
TOTAL		871.5	

FOS—fructo-oligosaccharides; RSD (%)—relative standard deviation; DP—degree of polymerization; LOQ—limit of quantification (0.018 mg for stachyose standard).

**Table 2:** Concentrations obtained for individual short-chain fatty acids (SCFAs), organic acids, and total SCFAs produced along fermentation time in fecal samples. The values are presented in g/L ± SD throughout time (h) and correspond to an average of the five donors.

SCFA (g/L)	Time (h)	Negative Control	Raftilose^®^ P95	Microbial-FOS	Statistical Significance
Acetate	0	0.05 ± 0.02	0.05 ± 0.02	0.05 ± 0.02	
	12	0.54 ± 0.16	0.83 ± 0.27	0.85 ± 0.29	a
	24	0.90 ± 0.10	1.48 ± 0.49	1.25 ± 0.32	a, c
Propionate	0	0.90 ± 0.19	0.90 ± 0.19	0.90 ± 0.19	
	12	0.30 ± 0.09	1.03 ± 0.13	1.25 ± 0.10	a, b, c
	24	0.44 ± 0.12	1.03 ± 0.13	1.30 ± 0.18	a, c
Butyrate	0	0.98 ± 0.04	0.98 ± 0.04	0.98 ± 0.04	
	12	0.88 ± 0.31	0.89 ± 0.44	1.18 ± 0.55	b
	24	0.73 ± 0.19	0.73 ± 0.12	1.22 ± 0.35	a, b
Lactate	0	0.25 ± 0.06	0.25 ± 0.06	0.25 ± 0.06	
	12	0.18 ± 0.04	0.87 ± 0.30	1.08 ± 0.12	a, c
	24	0.13 ± 0.05	1.33 ± 0.39	1.20 ± 0.42	a, c
Succinate	0	1.57 ± 0.27	1.57 ± 0.27	1.57 ± 0.27	
	12	0.53 ± 0.41	0.98 ± 0.40	1.37 ± 0.52	
	24	0.37 ± 0.26	0.66 ± 0.41	1.17 ± 0.51	a, b
Formate	0	0.42 ± 0.09	0.42 ± 0.09	0.42 ± 0.09	
	12	0.25 ± 0.06	0.31 ± 0.03	0.35 ± 0.05	
	24	0.18 ± 0.09	0.28 ± 0.08	0.35 ± 0.03	a
Total SCFAs	0	3.92 ± 0.18	3.92 ± 0.18	3.92 ± 0.18	
	12	2.50 ± 0.68	4.04 ± 0.48	5.00 ± 0.37	a, b, c
	24	2.62 ± 0.50	4.18 ± 0.76	5.29 ± 0.48	a, c

a, b, c: Statistically significant differences (*p* < 0.05) between a—Microbial-FOS and negative control; b—Microbial-FOS and Raftilose^®^ P95; c—Raftilose^®^ P95 and negative control.

## Data Availability

The data presented in this study are available on request from the corresponding author. The data are not publicly available due to restrictions e.g., privacy or ethics.

## Supplementary Materials

The following supporting information can be downloaded at: https://www.mdpi.com/article/10.3390/foods11070954/s1. Figure S1: Chromatogram obtained after derivatization in alditol acetates of the microbial-FOS mixture produced by the co-culture between A. ibericus and S. cerevisiae YIL162 W. (1) Internal standard (2-deoxyglucose); (2) mannose; (3) glucose; (4) sucrose; (5) 1-kestose; (6) neokestose; (7) nystose; (8) 1 F,6 G-Di-β-d-fructofuranosylsucrose or 6G(1-β-d-fructofuranosyl)2sucrose; (9) 1F-β-Fructofuranosylnystose; (10), (11) 1F-(1-β-d-fructofuranosyl)2-6G-β-d-fructofuranosylsucrose or 6G(1-β-d-fructofuranosyl)3sucrose or 1F-1-β-d-fructofuranosyl-6G-(1-β-d-fructofuranosyl)2sucrose. Table S1: Primer sequences targeting bacterial groups and qPCR conditions used for gut microbiota analysis. Adapted from Marques et al. [29]. Table S2: Glycosidic linkage composition of FOS mixture produced by A. ibericus co-cultured with S. cerevisiae YIL162 W. Table S3: Retention time, fragmentation pattern, possible assignment of oligosaccharides, and glycosidic linkage composition present in Raftilose® P95. Data were collected from Nobre et al. [23]. Table S4: Oligosaccharide composition expressed as the degree of polymerization of Raftilose® P95. Data were collected from Nobre et al. [23]. Table S5: Carbohydrate composition (mg/g) obtained for the microbial-FOS mixture produced by A. ibericus mono-culture.
