# Supplementary material for: Evaluation of Microbial-Fructo-Oligosaccharides Metabolism by Human Gut Microbiota Fermentation as Compared to Commercial Inulin-Derived Oligosaccharides
Source: Foods. 2022 Mar 25;11(7):954. doi: 10.3390/foods11070954 (PMC8997964; doi:10.3390/foods11070954)
Supplement: Supplementary file 1 [file foods-11-00954-s001.zip › foods-1628261-supplementary.pdf]

Supplementary Materials

Evaluation of microbial fructo-oligosaccharides metabolism by human gut microbiota fermentation as compared to commercial inulin-derived oligosaccharides

Dalila Roupar [1,2], Marta C. Coelho[1,2,3], Daniela A. Gonçalves[1,2], Soraia P. Silva[4], Elisabete Coelho[4], Sara Silva[3], Manuel A. Coimbra[4], Manuela Pintado[3], José A. Teixeira[1,2] and Clarisse Nobre[1,2*]

[1] CEB—Centre of Biological Engineering, University of Minho, Campus de Gualtar, 4710-057 Braga, Portugal; dalila.roupar@ceb.uminho.pt (D.R.); mcoelho@ucp.pt (M.C.C.); daniela.goncalves@ceb.uminho.pt (D.A.G.); jateixeira@deb.uminho.pt (J.A.T.)

[2] LABBELS—Associate Laboratory, Braga/Guimarães, Portugal

[3] Universidade Católica Portuguesa, CBQF—Centro de Biotecnologia e Química Fina — Laboratório Associado, Escola Superior de Biotecnologia, Rua Diogo Botelho 1327, 4169-005 Porto, Portugal; sara.nc.silva@gmail.com (S.S.); mpintado@ucp.pt (M.P.)

[4] LAQV-REQUIMTE, Department of Chemistry, University of Aveiro, 3810-193 Aveiro, Portugal; soraiapiressilva@ua.pt (S.P.S.); ecoelho@ua.pt (E.C.); mac@ua.pt (M.A.C.)

* Correspondence: clarissenobre@deb.uminho.pt; Tel.: +351-253-604-400; Fax: +351-253-678-986

Table S1. Primer sequences targeting bacterial groups and qPCR conditions used for gut microbiota analysis. Adapted from Marques *et al*. [29].

Target group	Primer sequence (5′-3′)	Genomic DNA Standard	PCR product size (bp)/ AT (°C)
Firmicutes	F: ATG TGG TTT AAT TCG AAG CA<br>R: AGC TGA CGA CAA CCA TGC AC	*Lactobacillus gasseri*<br>ATCC 33323	126/45
Bacteroidetes	F: CAT GTG GTT TAA TTC GAT GAT<br>R: AGC TGA CGA CAA CCA TGC AG	*Bacteroides vulgatus*<br>ATCC 8482	126/45
Bacteroides	F: ATA GCC TTT CGA AAG RAA GAT<br>R: CCA GTA TCA ACT GCA ATT TTA	*Bacteroides vulgatus*<br>ATCC 8482	495/45
Clostridium leptum subgroup	F: GCA CAA GCA GTG GAG T<br>R: CTT CCT CCG TTT TGT CAA	*Clostridium leptum*<br>ATCC 29065	239/45
Lactobacillus	F: GAG GCA GCA GTA GGG AAT CTT C<br>R: GGC CAG TTA CTA CCT CTA TCC TTC TTC	*Lactobacillus gasseri*<br>ATCC 33323	126/55
Bifidobacterium	F: CGC GTC YGG TGT GAA AG<br>R: CCC CAC ATC CAG CAT CCA	*Bifidobacterium longum* subsp. *infantis*<br>ATCC 15697	244/55

F—forward primer; R—reverse primer; AT—annealing temperature; bp—base pairs.

Table S2. Glycosidic linkage composition of FOS mixture produced by *A. ibericus co-cultured* with *S. cerevisiae* YIL162 W.

Glycosidic linkage	FOS mixture (mol %)	RSD (%)
t-Fru*f*	29.6	2
1-Fru*f*	41.8	7
6-Fru*f*	0.3	20
1,6-Fru*f*	0.4	28
**Total Fru*f***	72.2	
t-Glc*p*	24.3	10
4-Glc*p*	0.5	15
6-Glc*p*	3.0	6
**Total Glc*p***	27.8	

Table S3. Retention time, fragmentation pattern, possible assignment of oligosaccharides, and glycosidic linkage composition present in Raftilose® P95. Data was collected from Nobre *et al*. [23].

t_R[a]	Fragmentation pattern[b]	Possible assignment	Glycosidic linkages	FOS sample	
				Raftilose® P95	Microbial-FOS
28.68	153 (100); 375 (70.6); 211 (54.3); 169 (24.9); 128 (22.7); 109 (20.3); 433 (18.4)	Inulobiose [Fru(β2→1)Fru]	t-Fru; 1-Fru	+	+
29.04	153 (100); 375 (61.9); 211 (47.9); 169 (25.2); 128 (24.6); 109 (14.5); 433 (19.3)	[Fru(α2→6)Glc] / [Fru(β2→6)Glc]	t-Fru; 6-Glc	-	+
29.41	153 (100); 375 (69.2); 211 (49.3); 169 (21.8); 128 (27.0); 433 (20.6); 109 (12.1)	Inulobiose [Fru(β2→1)Fru]	t-Fru; 1-Fru	+	+
41.18	375 (100); 211 (75.0); 153 (60.3); 169 (47.1); 109 (26.0); 331 (25.0); 128 (15.8);	Inulotriose [Fru(β2→1)Fru(β2→1)Fru]	*t*-Fru; 1-Fru	+	-
41.38	211 (100); 169 (66.4); 109 (40.9); 331 (33.4); 101 (15.6); 127 (10.3); 128 (6.6);	[Fru(β2→6)Glc(α1↔β2) Fru] / [Fru(β2→6)Glc(α1↔α1) Glc] / [Glc(α1→6)Glc(α1↔β2) Fru]	t-Fru; 6-Glc / t-Fru; 6-Glc; t-Glc / t-Glc; 6-Glc; t-Fru	-	+
41.41	375 (100); 211 (72.2); 153 (53.7); 169 (41.0); 331 (23.3); 109 (23.0); 128 (13.8);	Inulotriose [Fru(β2→1)Fru(β2→1)Fru]	*t*-Fru; 1-Fru	+	-

a—Retention time (minutes) in the DB-1 column; b—Values in parentheses are the relative intensities of the fragments; (+)- present; (-)- absent; FOS – fructo-oligosaccharides.

Table S4. Oligosaccharides composition expressed as the degree of polymerization of Raftilose® P95. Data was collected from Nobre *et al*. [23].

Carbohydrate	**Sugar** (% ($g_{FOS}/g_{total\ sugar}$))				**Total FOS** (% ($g_{FOS}/g_{total\ sugar}$))
	DP3	DP4	DP5	DP6	
Raftilose® P95	33	30	20	5	95

DP—Degree of polymerization.

Table S5. Carbohydrate composition (mg/g) obtained for the microbial-fructo-oligosaccharides (Microbial-FOS) mixture produced by *A. ibericus* mono-culture.

	Assignment	FOS IB (mg/g)	RSD (%)
DP 1	Fru	5.1	8
	Glc	3.5	23
DP 2	Suc	69.5	23
	Inulobiose	36.0	29
	Blastose	11.5	20
DP 3	1-kestose	265.8	18
	Neokestose	22.2	5
	NR	6.3	11
	R	9.2	10
	R	5.0	6
	R	16.9	6
DP 4	Nystose	295.9	4
	1^{F},6^{G}-Di-β-D-fructofuranosylsucrose or 6^{G}(1-β-D-fructofuranosyl$)_2$-sucrose	39.9	5
DP 5	Fructo-furanosylnystose	24.6	31
TOTAL		811.5	

RSD (%)—Relative standard deviation; DP—Degree of polymerization; NR—non-reducing sugar; R—reducing sugar; FOS IB—Microbial-FOS.

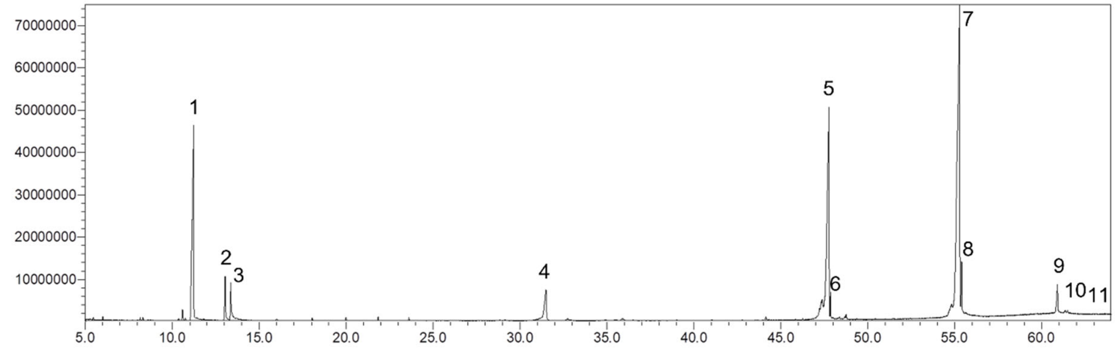

Figure S1. Chromatogram obtained after derivatization in alditol acetates of the microbial-FOS mixture produced by the co-culture between *A. ibericus* and *S. cerevisiae* YIL162 W. (1) Internal standard (2-deoxyglucose); (2) mannose; (3) glucose; (4) sucrose; (5) 1-kestose; (6) neokestose; (7) nystose; (8) 1^{F},6^{G}-Di-β-D-fructofuranosylsucrose or 6^{G}(1-β-D-fructofuranosyl$)_2$-sucrose; (9) 1^{F}-β-Fructo-furanosylnystose; (10), (11) 1^{F}-(1-β-D-fructofuranosyl$)_2$-6^{G}-β-D-fructofuranosylsucrose or 6^{G}(1-β-D-fructofuranosyl$)_3$-sucrose or 1^{F}-1-β-D-fructofuranosyl-6^{G}-(1-β-D-fructofuranosyl$)_2$-sucrose.
